# Tissue distribution of epirubicin after severe extravasation in humans

**DOI:** 10.1007/s00280-021-04280-8

**Published:** 2021-04-27

**Authors:** Jakob Nedomansky, Werner Haslik, Ursula Pluschnig, Christoph Kornauth, Christine Deutschmann, Stefan Hacker, Günther G. Steger, Rupert Bartsch, Robert M. Mader

**Affiliations:** 1 Division of Plastic and Reconstructive Surgery, Department of Surgery, Comprehensive Cancer Center of the Medical University of Vienna, Vienna, Austria; 2 Department of Medicine I, Clinical Division of Oncology, Comprehensive Cancer Center of the Medical University of Vienna, Währinger Gürtel 18-20, 1090 Vienna, Austria; 3 Department of Pathology, Comprehensive Cancer Center of the Medical University of Vienna, Vienna, Austria; 4 Department of Obstetrics and Gynecology, Comprehensive Cancer Center of the Medical University of Vienna, Vienna, Austria

**Keywords:** Extravasation, Surgery, Anthracycline, Tissue concentration, HPLC

## Abstract

**Purpose:**

As critical parameter after extravasation of cytotoxic vesicants, anthracyclines were determined in removed tissue from patients requiring surgical intervention due to tissue necrosis. We monitored their distribution within the affected lesion to establish a possible dose–toxicity relation.

**Methods:**

From six patients scheduled for surgery, removed tissue flaps were systematically analysed by HPLC (epirubicin: 5 subjects; doxorubicin: 1 subject).

**Results:**

After extravasation, tissue concentrations were highly variable with an individual anthracycline distribution pattern ranging from a few nanograms up to 17 µg per 100 mg tissue, which indicated a substantial difference in tissue sensitivity among patients. The resection borders coincided with the extension of the erythema and guided the surgical intervention after demarcation of the lesion, which occurred usually 2 or 3 weeks after extravasation. At that time, drug was hardly detected at the resection borders. Wound drains were negative for the extravasated drugs while showing a time profile of vascular growth factors and inflammatory cytokines, which was highly similar to routine surgery. In all six patients, surgical debridement with immediate wound closure led to healing within approximately 2 weeks, when therapy was resumed in all patients with reasonable time delay.

**Conclusion:**

Surgical intervention after demarcation of the extravasation lesion allows for almost uninterrupted continuation of treatment independent of the amount of extravasated anthracycline. As even minor amounts of the vesicants may trigger tissue necrosis, preventive measures merit the highest priority.

## Introduction

In contrast to many predictions, cytotoxics are still the cornerstone of cancer treatment on a global scale. With patients benefitting from novel therapeutic combinations, they have more lines of treatment available, which in turn enhances the likelihood of an extravasation event in the long run. Extravasation, a dreaded complication of chemotherapy, is still an elusive field with little evidence and guidance for individual management due to the high number of influencing variables [1]. Although anthracyclines are potent vesicants, it is not known if there is a subcutaneous tissue dose–response relation with a threshold level to initiate irreversible cellular damage resulting in tissue necrosis [2]. Despite efforts to refine and to detail management guidelines by cancer organizations [3], educational efforts [4] and deep literature search with emphasis on clinical evidence for prevention and management in cancer nursing [5], this topic was not addressed so far due to its experimental character. Moreover, little is known about the distribution within the affected lesion and the tissue clearance to reduce the toxic pressure and enable wound healing [6]. In one of the rare reports, tissue concentrations of platinum have been analysed at the anatomical level exploiting the high sensitivity of laser-coupled mass spectrometry [7]. To this aim, we have analysed tissue specimen from patients with anthracycline extravasation scheduled for surgical management due to tissue necrosis. This class of compounds was considered particularly suitable for this approach due to the high persistence of doxorubicin in tissue over weeks upon DNA binding in target cells combined with high chemical stability [8, 9]. Surgically removed tissue was divided into several specimens for chromatographic analysis to draw a map of local anthracycline concentrations centred around the injection site. This picture was completed by the analysis of anthracyclines and cytokines in wound drains over time.

## Methods

### Patient characteristics

Six patients, five women and one man, were enrolled in this study. Median patient age was 57 (45–78) years. In five of the six patients, the amount of the administered chemotherapeutic agent was documented (Table 1). The median amount of administered anthracycline was 133.8 mg (range 106.0–207.2 mg). Affected body sites were the forearm (3 cases), the cubital fossa (1 case), the dorsum of the hand (1 case) and the pectoral area (1 case). All patients were treated with surgical débridement followed by either split thickness skin grafting (3 cases) or local flaps (3 cases) for defect coverage.

**Table 1:** Demographics

Subject ID	Gender	Age	Cancer type	Administered drug	Dose administered
Patient 1	F	47	Breast	Epirubicin	134.58 mg
Patient 2	F	66	Breast	Epirubicin	127 mg
Patient 3	F	49	Breast	Epirubicin	207.16 mg
Patient 4	F	78	Breast	Epirubicin	106 mg
Patient 5	F	45	Breast	Epirubicin	133.76 mg
Patient 6	M	65	B cell lymphoma	Doxorubicin	Not documented*

* Patient 6 received the CHOP regimen with 50 mg doxorubicin/m^2^, but the exact dose could not be retrieved

The study was approved by the Institutional Review Board of the University Hospital Vienna and has, therefore, been performed in accordance with the ethical standards laid down in the 1964 Declaration of Helsinki and its later amendments.

### Initial conservative management

After the extravasation was noticed, administration of the chemotherapeutic agent was stopped immediately and all patients except for one, who was primarily treated at an external hospital, received topical treatment with dimethyl sulfoxide (99% pure) every 8 h, allowed to air-dry and followed immediately by local cooling over a period of at least 7 days (up to 14 days maximum). This was done according to the standard protocol in our institution. Due to the late discovery of more than 40% of extravasation events [10], the indication for dexrazoxane could be frequently not met (not mentioning other factors such as hospitalization and the toxicity of the antidote itself). In parallel, the affected area was immobilized and treated with a polyurethane foam dressing containing silver ions to prevent a secondary infection (MepilexAg, Molnlycke Health Care, Goeteborg, Sweden). Regular controls were performed every 3–5 days depending on the condition and severity of the lesion. The decision for surgery was taken after demarcation of the irreversibly damaged area within the lesion (centrally visible as necrotic tissue) encompassed by erythema. The surgical intervention was then scheduled within a median number of 10 days (range 1–19).

### Surgical management after demarcation

Surgeries were performed under general anaesthesia. The central skin necrosis and all of the encircling area showing erythema were resected including the deep fascia of the underlying muscle. The specimen was sent in for histological examination and high-performance liquid chromatography coupled to fluorometric detection. The tissue defects were covered either by split thickness skin grafts or tissue flaps (muscle or fascio-cutaneous). Split thickness skin grafts were covered with negative pressure wound therapy systems. Regular drains were used in tissue flaps. Postoperative drainage fluid was collected and analysed as well.

### Analysis of anthracyclines by high-performance liquid chromatography (HPLC)

Anthracyclines were analysed using an HPLC method coupled to fluorescence detection as described previously [11]. During surgical intervention, samples were collected from wound drains and from resected pathologic tissue specimen. Briefly, drain samples were centrifuged before injection into a HPLC system (Agilent series 1100, Germany), whereas tissue specimen were homogenised in 10% dimethyl sulfoxide in physiologic saline in a gentleMACS Dissociator (Miltenyi Biotec, Germany) according to the manufacturer’s instructions before injecting the centrifuged supernatant. HPLC conditions for the isocratic elution of epirubicin were 0.1% triethylamine in water (adjusted to pH 2 with trichloroacetic acid) and acetonitrile (68:32) at a flow rate of 1 ml/min on a LiChrospher 60 RP-select B column (5 mm particle size, from Merck, Germany) coupled to fluorescence detection (excitation: 478 nm; emission: 565 nm). The limit of detection was 1.03 ng epirubicin/100 mg tissue with a limit of quantification of 1.72 ng epirubicin/100 mg tissue defined as signal:noise ratio = 5:1. Samples were stored at −30 °C until analysis.

## Results

In this study, we investigated six subjects after extravasation of an anthracycline scheduled for surgical intervention. Based on this criterion, patients were heterogeneous in their demographics, although most of the patients had breast cancer (Table 1). Likewise, the anatomical location and dimension of the affected area differed among patients (Table 2). Surgery was performed with a median time frame of 32 days after extravasation (range 21–54 days) as surgery took place after demarcation of the affected area to identify necrotic tissue. The median hospital stay after surgery was 12.5 days (range 8–16 days). With the exception of subject 5, the necrotic area of enrolled patients prior to surgery was below 10% of the dimension of erythema. It was, however, much larger with 38% in the pectoral region in subject 5.

**Table 2:** Extravasation characteristics

Subject ID	Affected site	Days until surgery	Size skin erythema (cm)	Size skin necrosis (cm)	Defect coverage	Hospital stay (days)
Patient 1	Forearm	38	5 × 7	0.8 × 0.7	Skin graft	8
Patient 2	Cubital fossa	54	5 × 5	1 × 1	Brachioradialis flap	11
Patient 3	Forearm	23	13 × 7.5	1.5 × 1.5	Skin graft	13
Patient 4	Dorsum of the hand	46	4 × 4	1 × 1	Radial forearm flap	12
Patient 5	Pectoral region	21	14 × 6	8 × 4	Latissimus dorsi flap	16
Patient 6	Forearm	26	13.5 × 10	5 × 2	Skin graft	16

Tissue concentrations differed substantially among patients ranging from a few nanograms up to 17 µg per 100 mg tissue (Table 3). Despite this high variability, some observations seem to apply in general. First, even in subjects with high tissue concentrations, there were regions without detectable anthracycline or only trace amounts of the drug. Second, the centre of the necrotic region was not necessarily the specimen with the highest drug concentration.

**Table 3:** Individual anthracycline concentrations in tissue (ng anthracycline/100 mg tissue)

Subject ID	Minimum conc.	Maximum conc.	Central conc.
Patient 1	0	25	25
Patient 2	0	12	0
Patient 3	0	1402	0
Patient 4	1	3007	244
Patient 5	3	104	25
Patient 6	0	17,451	11,819

*conc.* concentration; central concentration refers to the specimen collected at the centre of the necrosis

**Fig. 1:**
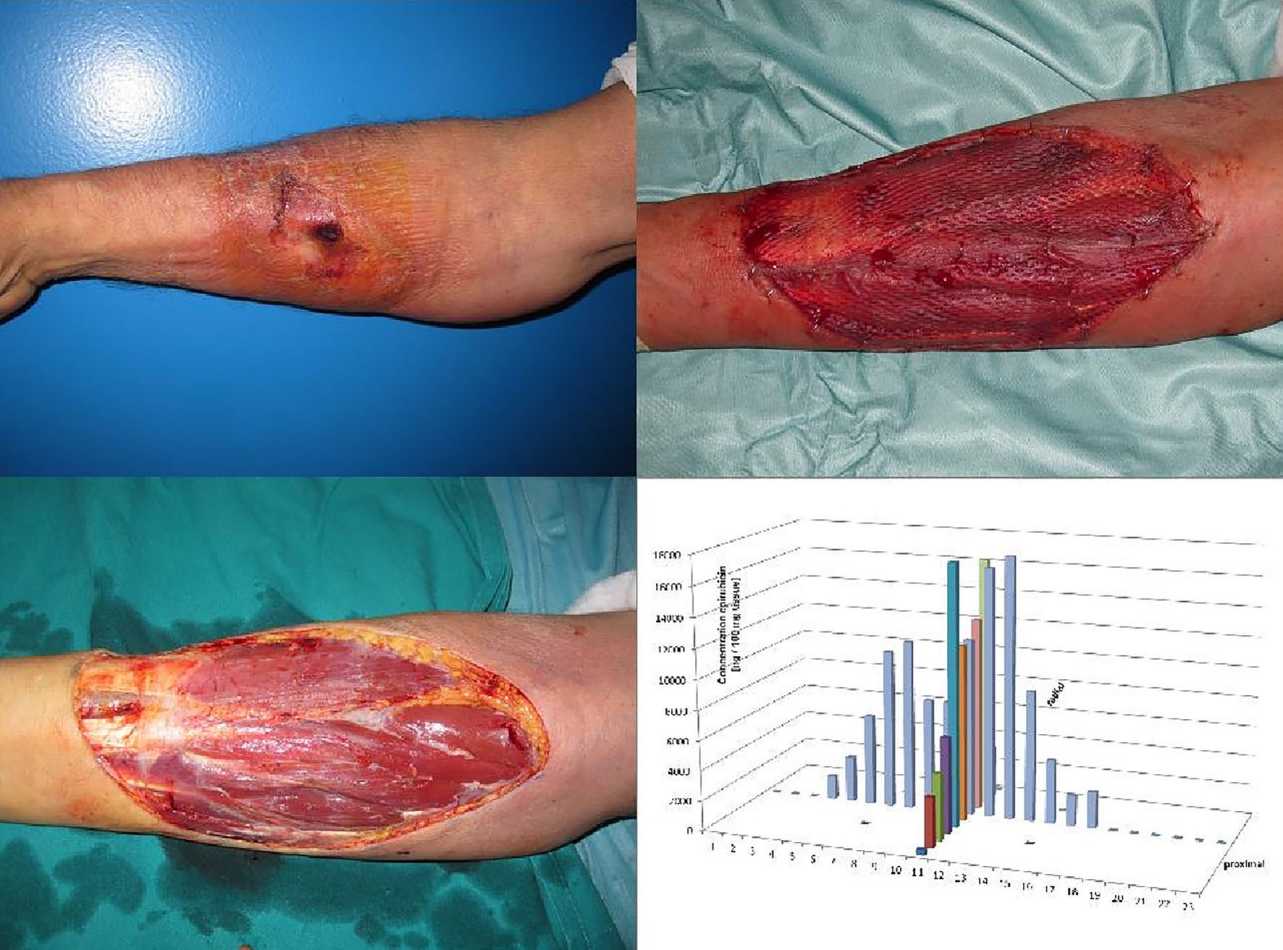
Doxorubicin tissue distribution after extensive extravasation (subject 6). A 65-year-old male lymphoma patient suffered a doxorubicin extravasation on the right forearm (upper left picture). Surgery was performed 26 days after the incident (lower left picture). The defect was covered with a split-thickness skin graft (upper right picture). HPLC analysis showed a pyramid-shaped distribution pattern with the highest concentrations in the central area, where the extravasation originally happened (lower right picture)

**Fig. 2:**
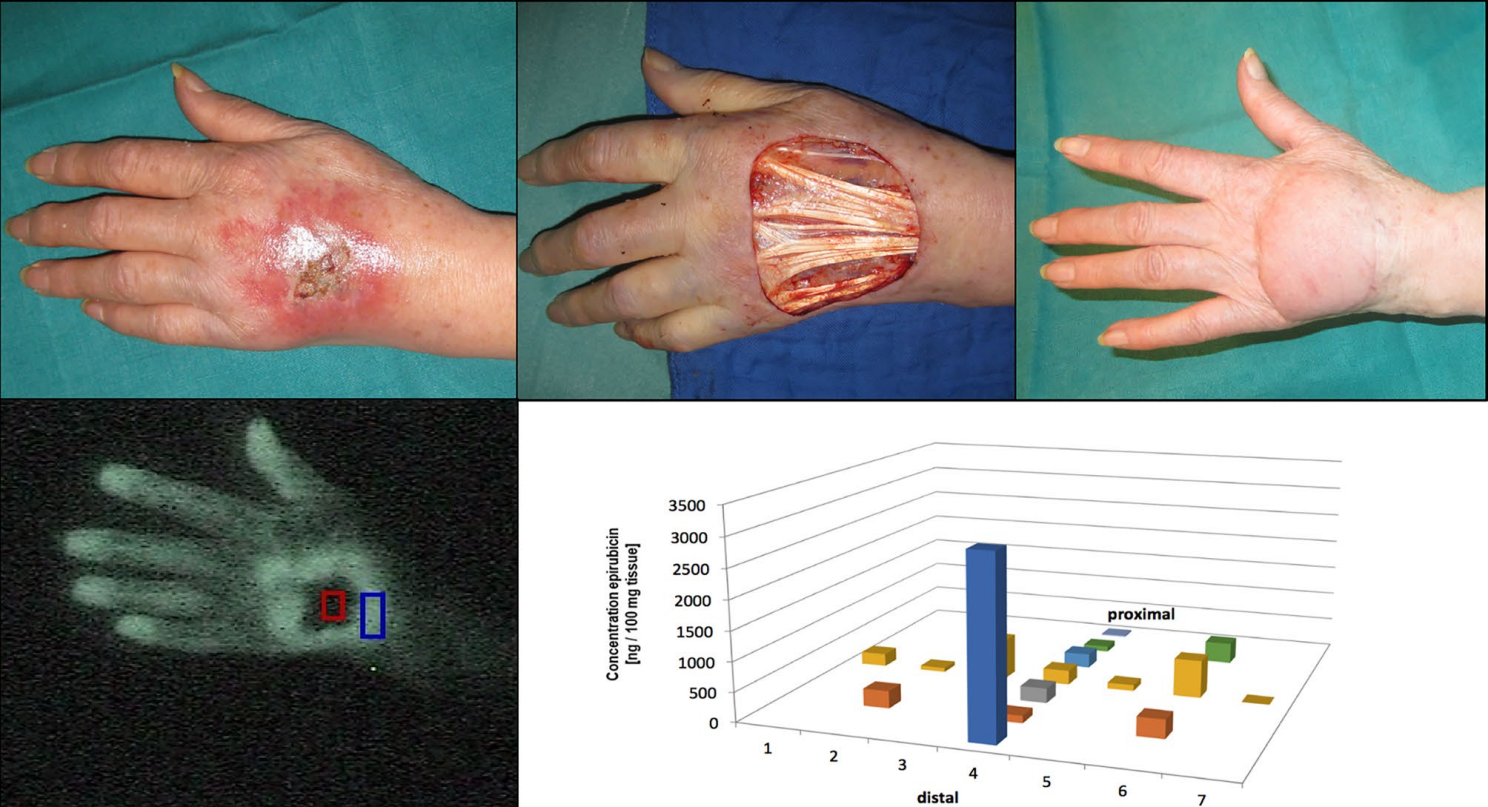
Epirubicin extravasation with substantial tissue distribution (subject 4). A 78-year-old female breast cancer patient suffered an epirubicin extravasation on the dorsum of her left hand. After demarcation, surgery was performed 46 days later. The defect was covered with a radial forearm flap. Indocyanine green video angiography immediately after the extravasation event (lower left picture) already predicted the central necrosis (dark area) and the hypervascularized erythema area (white area). HPLC analysis of the resected tissue showed the highest concentration of epirubicin in the most distal part, most likely due to the effects of gravitation affecting the distal extremities (lower right picture)

To illustrate the tissue distribution within a lesion, two subjects have been chosen, the subject with the highest measured amounts of extravasated drug and an extensive extravasation as judged by the area of the affected lesion (Fig. 1) and one subject, where the drug has probably diffused away from its original position leaving the central part of the lesion with small amounts of epirubicin (Fig. 2).

The patient shown in Fig. 2 was one out of three subjects with detectable amounts of drug at the resection border, whereas the other three patients followed the pattern shown in Fig. 1. Despite this, there were no complications in wound healing after surgery, which allowed resuming systemic treatment in all subjects. To emphasise these observations, wound drains were collected up to 5 days after surgical intervention to determine if residual drug was still washed out from the wound bed. In all six patients, wound drains were negative for anthracyclines throughout the whole observation period.

In parallel, wound drains were analysed for the presence of vascular growth factors and inflammatory cytokines. These profiles were compared with that monitored in patients undergoing routine surgery without cytotoxic treatment. In line with the absence of drug in wound drains, the time profiles of vascular growth factors (vascular endothelial growth factor, platelet-derived growth factor and angiopoietin) and the inflammatory cytokine interleukin-33 did not differ from the drain kinetics after routine surgery in healthy subjects.

As a consequence, one would expect to resume systemic treatment and to continue cancer chemotherapy within a reasonable time frame with minimal delay for the next therapeutic cycle. This corroborates the chosen management approach despite the limited number of observations.

In conclusion, extravasation was associated with a very high individual variability ranging from a few nanograms of anthracycline to micrograms in tissue, covering three orders of magnitude. Even very small amounts of the vesicant may be able to cause necrosis with the need for surgery. The surgical intervention after demarcation of the lesion was able to remove the drug as judged by the rapid wound healing, the absence of extravasated drug in wound drains resulting in the re-start of systemic treatment within a reasonable time frame.

## Discussion

The patients investigated in this study showed a highly individual anthracycline distribution pattern after extravasation combined with a substantial difference in tissue sensitivity. Interestingly, the central part of the tissue removed during surgery, which was irreversibly damaged (full-thickness necrosis) in all subjects, was not always the area with the maximum anthracycline concentration. This pattern suggests that even small amounts of anthracyclines may act as a vesicant in subjects with high sensitivity to the cytotoxic in the subcutaneous tissue. This initial sensitivity may then be further aggravated by the impaired blood flow in the central part of the lesion as demonstrated by our group [12]. The hypoemic conditions within the lesion—observed in all three investigated patients of this cohort—result in hypoxic conditions thus fuelling tissue collapse. Moreover, anthracyclines are known to be stable in highly persistent in tissue as they are DNA intercalating agents and are released upon cell death. Subsequently, they may be incorporated in neighbouring cells, which drives a vicious cycle eventually leading to necrosis [8, 13]. As indocyanine green angiography was correctly predicting necrosis in all three examined patients, the central role of continuous blood flow as drug clearing mechanism from the affected lesion is emphasised with deleterious effects in combination with vesicants.

In previous studies with surgical management of extravasation injuries, the surgical approach and the timing of surgery were variable. Some authors resected only the necrotic tissue, whereas others resected the whole area which was macroscopically affected by the extravasation [14, 15]. Concerning the timing for the surgery, some authors recommend early debridement, whereas others preferred delayed debridement [16]. In previous studies with surgical management of extravasation injuries, the surgical approach and the timing of surgery were variable [11]. The surgical approach for the patients in this study was radical debridement of all tissue that was macroscopically affected by the extravasation after demarcation of the affected lesion. This approach did not only include areas with necrosis, but also areas with erythema. The goal was to eliminate the cytotoxic vesicant completely during one surgical intervention to create favourable conditions for successful defect coverage. In addition, minimising the risk for wound healing problems was supposed to reduce the delay for resumption of the next therapeutic cycle. Whenever suitable, surgeries were, therefore, scheduled shortly after a therapeutic cycle to provide the maximum time frame for complete wound healing before the next therapeutic cycle was scheduled. As surgery was performed with little variation approximately 3 to 6 weeks after extravasation in this study, a significant impact of timing on tissue levels of anthracyclines seems unlikely considering also the low tissue clearance of this class of compounds.

Further evidence for this approach came from the results of the HPLC analysis in the surgically removed tissue, which demonstrated the presence of the cytotoxic vesicant in areas with erythema. The absence of bacterial colonization but also that of inflammatory signals is of importance for successful graft take in skin transplantations ensuring defect coverage [17, 18]. Wound drains analysed in this study did not show elevated growth factor or interleukin-33 levels when compared with drains from conventional wound surgery. This fact in combination with the short postoperative healing times after skin grafting seems to support the chosen approach [10]. The absence of elevated inflammatory signals in the wound drains also questions the use of corticosteroids as intervention, which has been recommended in the past and is still is widely accepted despite discouraging results in animal studies and little evidence from histopathological studies, where migration of inflammatory cells in the affected lesions is a secondary event [19–21].

Limitations of this investigation are the lack of information about the amount of extravasated drug, which would be helpful in the interpretation of a possible dose–toxicity relation. So far, there are no objective approaches to collect this valuable information as extravasation events are frequently detected only one or more days later [10]. Another limitation was that anthracyclines are converted to several known metabolites in the systemic circulation including the most relevant one doxorubicinol, whereas over time glucuronides are formed in blood to facilitate excretion. If present in tissue, however, we would have detected the main metabolite doxorubicinol in tissue samples and wound drains, which was, however, not the case. This at least suggests poor tissue metabolism of the drug, which is in line with high tissue persistence and low clearance of the drug from peripheral compartments.

In summary, anthracyclines initiate a vicious cycle upon extravasation with primary cellular damage, hypoemic conditions leading to hypoxic conditions with fast tissue necrosis. This effect may be caused already by very small amounts of extravasated anthracycline emphasising the role of prevention. Five of the six study patients experienced extravasation in body parts (hand, forearm, cubital fossa) of great importance for successfully managing daily life tasks. Skin grafts and local flaps, which were used for defect coverage after radical debridement, led in all cases to highly satisfying functional and aesthetic postoperative results with acceptable time delay for the next therapeutic treatment cycle. However, these investigations demonstrate that only strict intravasal application prevents tissue damage after application of anthracyclines, which may cause necrosis in the nanogram range. This study, therefore, provides experimental evidence why prevention is the most critical step when administering anthracyclines to avoid a dreaded complication.

## Data Availability

The data sets generated in this study are sensitive data and subject to data protection. Therefore, they are available with some restrictions from the corresponding author on reasonable request in compliance with personal data regulation policies.
